# Research gaps in transforming tuberculosis data to action for better health outcomes: A systematic literature review

**DOI:** 10.7189/jogh.11.04058

**Published:** 2021-09-18

**Authors:** Manish Kumar, Meredith Silver, Jeanne Chauffour, Colleen Boyle, David Boone

**Affiliations:** 1 Carolina Population Center, University of North Carolina at Chapel Hill, NC, USA; 2 Public Health Leadership Program, University of North Carolina at Chapel Hill, NC, USA; 3 John Snow Inc., Washington, D.C., USA

## Abstract

**Background:**

In their work to end the tuberculosis (TB) epidemic in lower- and middle-income countries, national TB programs need a tool to measure, monitor, and strengthen relevant capabilities to create a continuous transformation of data into action (D2A) to improve TB program results. However, there is a lack of scientific evidence to determine specific measurement dimensions of a D2A continuum that enables TB programs to identify the barriers and enablers of D2A and to guide the selection of interventions appropriate for the context and decision-making capabilities of various TB program actors.

**Methods:**

A systematic review of peer-reviewed and grey literature was conducted per Preferred Reporting Items for Systematic Reviews and Meta-Analyses guidelines. A total of 48 peer-reviewed publications were selected for data extraction and analysis.

**Results:**

The findings show that selected publications discussed the health system level, stakeholders involved in decision making, decision type, data system, data sources, data use enablers and barriers, decision-making framework and steps, decision outcome/impact, and how that outcome was measured. The findings highlight barriers and enablers to data use and explain the relationship among data sources, decision type, and stakeholders. Seventeen D2A measurement dimensions were identified.

**Conclusions:**

Transforming data to action is a continuous process that recognizes that data use indicators vary by type of decisions, decision makers, and the health system level at which decisions are made. As a logical next step, the project team plans to develop and validate a D2A continuum toolkit that will include a measurement scale, implementation guide, and data collection and analysis Microsoft Excel workbook.

The demand on national tuberculosis (TB) programs for data are driven by the need to better target TB programming to reach people and identify missing cases; to improve the quality and efficiency of TB services; to effectively plan and advocate for resources; and to hold national TB programs and institutions accountable for resource use and results. As such, the monitoring and evaluation (M&E) and surveillance systems of national TB programs need to be fully functional and be able to address the many data demands of stakeholders, including health ministry officials, district and facility managers, individual providers, legislative bodies, communities, and the media. However, many countries have inadequate systems in place. M&E and surveillance systems for TB face some of the same generic challenges of any health information system (HIS): high-quality data are not routinely collected with sufficient detail to allow regular computation of levels, trends, and inequalities that affect the ability of programs to use the data for implementation at national and local levels. TB M&E and surveillance systems also have the challenges of effective integration of various components of the routine information system, such as service statistics, disease surveillance, financial and human resource resources data, etc. This integration is necessary to show the performance of the TB program in successfully providing TB services and to capture the commitment of the program for that progress. A country’s progression along the TB data-information-knowledge-action (ie, data to action) continuum provides national policymakers, development partners, civil society (including faith-based and local organizations), and the private sector with information on where to invest in a select number of interventions and tools that synergistically have the greatest impact on the quality, availability, analysis, use, and accessibility of TB data. “Data to action” refers to databased decision making that shows a measurable impact of improvements in processes (eg, data collection processes), systems (eg, HIS), human resources (eg, health workers, managers), and institutional attributes (eg, policy, strategy, governance) associated with national TB program goals.

The Global Accelerator to End Tuberculosis (Accelerator) model of the United States Agency for International Development (USAID) aims to build local commitment and capacity to support countries in reaching global targets of diagnosing and enrolling 40 million people on TB treatment and 30 million on TB preventive therapy by 2022 [1]. Key to achieving these ambitious goals and to monitoring implementation of the Accelerator is ensuring that countries have an effective system for data collection, analysis, and use. Many resources (tools, methods, frameworks) exist for improving data use for strategic and programmatic purposes; however, they are not always well adapted to local needs [1,2]. The selection of an appropriate approach or tool, adapted to the country context, is imperative for effective interventions to transform data to action [3]. Each country and program are unique, making it difficult to choose the right interventions targeting decision makers at various levels. Moreover, to monitor implementation of the Accelerator and ensure that countries use data for decision making, their data use capabilities must be measured, monitored, and strengthened in accordance with the Accelerator’s Performance-Based Monitoring and Evaluation Framework (PBMEF) [4]. The PBMEF is a key component of the Accelerator’s efforts to ensure effective accountability and monitoring of progress in reaching specific country and global TB milestones and targets. Increased capability to transform data to action would enable national TB programs to analyze, visualize, and use TB data throughout the decision-making process. Countries need a tool that identifies the characteristics at each stage of the process to continuously transform TB data to action.

However, there is lack of scientific evidence to determine specific measurement dimensions of a data to action continuum for TB data use that enables national TB programs to precisely gauge the barriers and enablers to data use and to select interventions that are appropriate for both the program context and the decision-making capabilities of different actors across levels. Therefore, a systematic review of available scientific literature and country-specific policy, program, or strategy documents was conducted to answer two questions:

1. What are the barriers and enablers to transforming TB data to action in the context of lower- and middle-income countries (LMICs)?
2. What are the appropriate measurement dimensions to gauge national TB program’s capabilities to transform data to action?

Because the preliminary analysis showed a paucity of peer-reviewed literature focusing on TB data use across the health systems levels in the LMICs, the scope was broadened to draw lessons from other health system areas. The answers to these questions will help in the design of a data to action continuum (D2AC) tool.

## METHODS

A two-pronged strategy was applied that included reviews of peer-reviewed and grey literature (not peer reviewed for a publication) and consultation with TB experts. For the peer-reviewed literature portion, a comprehensive survey was conducted using scientific electronic databases. The grey literature search was conducted in electronic repositories to identify relevant documents (eg, project reports, policy and program documents, technical publications, M&E reports). A group of experts was also consulted to identify relevant peer-reviewed and grey literature for inclusion.

### Comprehensive literature search and review

#### Peer-reviewed literature search and review

A systematic and focused search used search terms based on input from the USAID-funded TB Data, Impact Assessment and Communications Hub (TB DIAH) project team and in consultation with a health informatics librarian at the University of North Carolina (UNC) at Chapel Hill. The electronic search did not limit results by publication date or geography but was restricted to English-language publications. Because test search results showed a limited number of TB-specific peer-reviewed articles, a broader strategy was needed that aimed to draw lessons from the literature on health systems and health care.

An initial test electronic search was performed on June 13 and June 25, 2020, across four scientific databases: PubMed, Web of Science, SCOPUS, and Global Health. The purpose was to review preliminary results and validate the search terms. The database search was completed in July-August 2020, using search terms related to health care delivery, HIS, data use, decision making, and low resource settings. (A complete list of search terms is given in Table S1 of the **Online Supplementary Document**). References (n = 132) from two of the systematic review papers included in the screening process were extracted from SCOPUS and added to the publication screening list in Mendeley. Five additional papers were identified through professional networks and experts and were added to the screening list.

#### Grey literature search and review

This search included electronic health repositories of the World Health Organization (WHO) headquarters and its regional offices in Africa and the Americas, the USAID-funded MEASURE Evaluation and Health Policy Plus projects, and the Health Data Collaborative, among others (Table S2 in the **Online Supplementary Document**).

The expert organizations with international and country-level experience in TB programs and data systems that were consulted to identify relevant publications are listed in **Table 1**.

**Table 1:** List of organizations with experts that were consulted to collect grey literature

No.	Organization name	Country
1	WHO	Switzerland
2	Global Fund to Fight AIDS, Tuberculosis and Malaria	Switzerland
3	UK Aid Direct	United Kingdom

### Data management

The Mendeley/Covidence reference management system was used to organize, clean duplicates, and complete the screening of abstracts and full-text reviews.

### Inclusion and exclusion criteria

Peer-reviewed publications focused on data use barriers and enablers, actors, levels, and decision types (operational, clinical, policy, programmatic, etc.) in health care systems of LMICs were included for title and abstract screening. If abstracts were unavailable for grey literature documents, executive summaries and tables of contents were reviewed. All non-English-language papers and those describing the developed country context were excluded, as were conference posters, abstracts, and presentations. The same screening criteria were used for the full-text review; papers were excluded if the full text was unavailable. Country-level documents (evaluation reports, technical reports, policy documents, etc.) describing TB data flow, data quality, data use, and decision making were included if available in the public domain and published in English.

### Document screening and data extraction

A three-member team completed the title and abstract screening and full-text reviews for the peer-reviewed papers. Two members independently completed the title and abstract screening; conflicts were resolved through consultation. Two members independently conducted full-text reviews of the same set of five papers. The purpose was to standardize the review parameters before completing all full-text reviews. A third team member helped resolve conflicts associated with inclusion or exclusion of the full-text paper. The grey literature was reviewed by a separate team of two members and any conflict was resolved in discussion with the peer-reviewed literature screening team. A Microsoft Excel-based (Microsoft Incl, Seattle, WA, USA) data extraction template was created and tested. Two team members extracted data for two papers to test this template. The entire team reviewed the extracted data, and added instructions and clarified terms to standardize the template. The document screening and data extraction were completed during August-October 2020.

### Data analysis

Extracted data were analyzed using both qualitative and quantitative techniques. The framework approach [5] was adapted to analyze qualitative data and identify appropriate themes (here called dimensions). Data analysis in the framework approach includes a series of interconnected stages that enable the researcher to move back and forth across the data until a coherent account emerges [6]. This approach allowed us to continuously refine the data to action measurement dimensions we sought to identify through the literature review. **Table 2** provides a quantitative summary of the scientific database search results.

**Table 2:** Scientific database search results (as of July 1, 2020)

Order	Database name	Number of results
1	SCOPUS	122
2	Web of Science Core Collection	42
3	PubMed	56
4	Global Health	160

**Figure 1:**
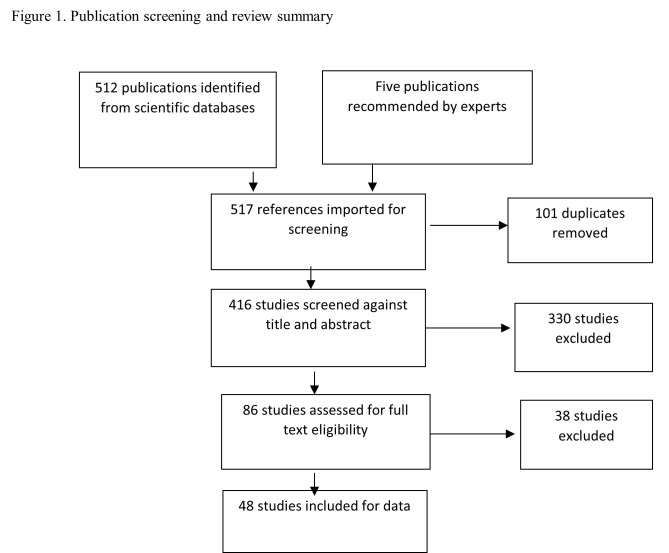
Publication screening and review summary.

References (n = 132) from two of the systematic review papers were extracted and included in the screening list. The database search resulted in 512 publications, and a curated list of five publications was recommended by international experts. The title and abstract screening included 416 publications after 101 duplicates were removed. The title and abstract review resulted in the selection of 86 publications for full-text reviews. A total of 330 abstracts were excluded because they did not focus on data use or decision making in the context of LMICs, and were conference posters, abstracts, and presentations. A total of 48 publications (Table S3 in the **Online Supplementary Document**) were ultimately selected for data extraction, review, and analysis (**Figure 1**). The 38 excluded papers did not focus on data use or decision making, whereas the rest of the papers focused on technology adoption, program evaluation, prototype design, workforce intervention for monitoring, theory for HIS decentralization, and benefits of computerized league tables. The grey literature analysis focused on policy, program, strategy, and project documents describing TB data use, data systems, and decision making at national and subnational levels. Table S4 in the **Online Supplementary Document** lists the grey literature that was included. Table S5 in the **Online Supplementary Document** lists the HIS-related assessment tools that were included in this review.

## RESULTS

This section describes the findings in terms of the distribution of papers (by decision type, level of health care, geographical focus), barriers and enablers for data use, and linkages among data sources, decision type, and stakeholders.

### Publication focus and distribution

The studies identified for review discussed the level of the health system, stakeholders involved in decision making, decision type, data system, data sources, data use enablers and barriers, decision-making framework and steps, decision outcome/impact, and how that outcome was measured.

The data extraction and analysis were guided by clinical, operational, program, and strategy/policy decision making. **Table 3** shows the decision types and the definitions used.

**Table 3:** Types of decisions and their definitions

Type of decision	Definition
Clinical	Decision making associated with individual patient care; includes all types of health workers
Operational	Decision making related to infrastructure, workforce, funding, and training required to support clinical, program, and strategic decisions
Program	Decision making related to program planning, development, and M&E targeting population, community, or individual health care program interventions
Strategy/Policy	Decision making focused on creating the overarching framework and/or guiding principles for the development and implementation of programs to achieve both population- and patient-level health outcomes

**Table 4** summarizes the findings by number of publications in each decision type category: clinical, operational, program, and strategy/policy. A review of all 48 studies shows that most publications (19) discussed strategic/policy decisions, followed by operational (11), program (3), and clinical (3) decisions. Five studies mention more than one type, whereas 24 provided decision-making frameworks that were implemented in the decision-making process in the country studied. Some studies mentioned multiple types of decisions and are therefore counted multiple times. The table also shows the number of publications describing each health system level and by geographical focus, with the largest number focused on Tanzania (9), followed by Ethiopia (7), India (5), and Malawi (4). Five papers named LMICs as their geographical focus.

**Table 4:** Summary table of included peer-reviewed studies (n = 48)

Type of decision	# of publications
Strategic/policy	19
Operational	11
Program	3
Clinical	3
**Level of health care**	**# of publications**
General	29
National	11
Subnational (state, regional, provincial)	9
District	20
Facility	16
Clinic	5
Community	9
**Top five geographical focus areas:**	
Tanzania	9
Ethiopia	7
Other LMICs	5
India	5
Malawi	4

In terms of health domain, three publications focused specifically on TB, five on HIV, and one on malaria, whereas the remainder focused specifically on HIS or HIS for maternal and child health programs.

### Data use barriers and enablers

Several publications highlighted barriers to and enablers of data use at different health system levels. Table S6 in the **Online Supplementary Document** summarizes the barriers reported in eight of the papers as examples, which included low-quality data, shortage of data collection and reporting tools, lack of data analysis skills, substandard M&E, poor Internet connectivity, and rampant redundancies in HIS [7].

Table S7 in the **Online Supplementary Document** presents the data use enablers reported in eight of the papers as examples, which varied by country and health system level. Key data use enablers included data collection in alignment with workflow, established analytics and visualizations, robust standard operating procedures, strong presence of data governance, increased data quality, rapid data transmission, regular and timely feedback to facilities, and use of HIS at the community level.

Selected key data use barriers and enablers reported in the grey literature are presented in Table S8 in the **Online Supplementary Document**. They are similar to those identified in the peer-reviewed literature but they have wider applicability, given the global focus of the reports/publications.

### Relationship among data sources, decision type, and stakeholder

As shown in the previous tables, decision type varied by health system level, and data use barriers and enablers varied by both health system level and country context. Table S9 in the **Online Supplementary Document** presents summary findings describing the relationship among data sources, decisions, and stakeholders involved in decision making.

In most situations, multiple stakeholders are involved in collecting and sharing data at different levels, irrespective of the decision type. However, clinical and health facility data sources are primarily used by providers and managers for clinical and managerial decisions. For example, Avan, Berhanu, Umar, Wickremasinghe, and Schellenberg (2016) reported that primary and secondary health care data were used by providers at the facility and district levels [8]. In another example, Findikoglu and Watson-Manheim (2016) reported that primary care providers used electronic health records for decision making [9].

### Data to action measurement dimensions

Using the framework approach to qualitative data analysis [5], a set of 17 data to action measurement dimensions were identified and defined (**Table 5**). These dimensions respond to the research question that aimed to identify appropriate dimensions required to improve national TB program capabilities for transforming data to action.

**Table 5:** Data to action measurement dimensions and definitions

Dimension	Definition
**Data collection tool and workflow**	The tools/devices/instruments used as data sources for ongoing systematic data collection and reporting to support analysis, interpretation, storage, and sharing of TB program data.
**Data quality**	The completeness, timeliness, consistency, reliability, and accuracy of data.
**Data integration and exchange**	The mechanism for transforming and integrating data from multiple sources in a target destination environment; can also refer to the activities of matching, merging, and deleting records in a single data store.
**Analytics and visualization**	The use of analytics and visualization techniques and decision support tools to provide new insights and patterns from data analysis to stakeholders at different levels to enhance health and health care decision making.
**Data synthesis and communication**	How data are processed, analyzed, and presented in appropriate visualizations, and can be understood and used by the target audience.
**Data use policy**	The process, procedures, and actions of an organization associated with the collection and sharing of its data.
**Data governance**	The development and implementation of administrative policies, procedures, and processes that define workflow, program inputs and outputs, management structure and oversight functions, and the methods and frequency of performance evaluation.
**Data access and sharing**	The disclosure of data from one or more organizations to another organization(s), or the sending of data between different parts of a single organization. This can take the form of routine data sharing, where the same data sets are shared between the same organizations for an ongoing established purpose, and exceptional, one-off decisions to share data for a specific purpose.
**Organizational structure and function**	The organizational structures and processes, including job titles and clear descriptions of duties and responsibilities.
**Leadership and coordination**	The exercise of technical, political, and administrative authority to manage the national TB program at all levels of a country’s health system. The leadership and coordination structure consists of the mechanisms, processes, and institutions through which actors and stakeholders articulate their interests, exercise their rights, meet their obligations, mediate their differences, and oversee the performance of the TB program.
**Monitoring, evaluation, and learning**	A plan supporting management of program activities and informing the organization about what activities to implement, timeline, resources, responsible party, and whether and how an activity is contributing to stated program goals.
**Financial resources**	The legal and administrative systems and procedures in place that permit a government ministry and its agencies and organizations to conduct activities that ensure the correct use of public funds and that meet defined standards of probity and regularity. Activities include management and control of public expenditures, financial accounting, reporting, and asset management, in some cases.
**Data use capacity**	The organizational structure and individual ability that enable data users to read, write, and communicate data in context, including an understanding of data sources and constructs, analytical methods, and techniques applied; and the ability to describe the use case, application, and resulting value.
**Health workforce capacity development**	The development of a skilled, flexible, and diverse workforce that meets the current and expected needs of the national TB program.
**Hardware**	An assembly of tangible physical parts of a system of computers, including servers and virtual private networks, which provide services to a user in the health information ecosystem.
**Network and Internet connectivity**	Network is the disparate elements of a system connected in a way that data and information can be shared among all elements. Internet connectivity refers to how people are connected with computers and other digital devices for near instantaneous access to data and information.
**ICT infrastructure**	An ICT infrastructure refers to a necessary condition required for the development, deployment, and use of information technology services, managing, and sharing quality data among systems.

**Table 6** summarizes the number of publications describing barriers and/or enablers for each dimension listed in **Table 5****.** The top three reported enablers to data use are leadership and coordination (19), health workforce capacity development (18), data collection tools and workflow (17), and data quality (17). The top three reported barriers to data use are lack of health workforce capacity development (48), poor data quality (37), and siloed data collection tools and workflow (30).

**Table 6:** Publications describing data use enablers and barriers for each data to action dimension (from peer reviewed literature [n = 48])

Data to action dimension	Number of peer-reviewed publications in data use enablers group	Number of peer-reviewed publications in data use barriers group
Data collection tool and workflow	17	30
Data quality	17	37
Data integration and exchange	14	8
Analytics and visualization	11	10
Data synthesis and communication	16	14
Data use policy	4	8
Data governance	15	8
Data access and sharing	13	9
Organizational structure and function	9	7
Leadership and coordination	19	19
Monitoring, evaluation, and learning	10	6
Financial resources	2	12
Data use capacity	6	11
Health workforce capacity development	18	48
Hardware	5	7
Network and connectivity	4	10
ICT business infrastructure	11	16

## DISCUSSION

National TB programs in LMICs are investing in HIS to produce higher quality and more timely data. Research shows that data collection and data availability are not sufficient to ensure that data are used for program planning, policy development, and clinical care. Data-informed decision making is the outcome of complex system dynamics in which technical, organizational, and behavioral dimensions interact to create specific enablers or barriers to data use [10,11]. Transforming data to action is a continuous process whose metrics need to reflect data use characteristics across a continuum [3] of decisions. Yet there is a lack of understanding about the interactions among national TB program stakeholders, data systems and workflows, and decision making, and how such interactions impact improvements in data use capabilities at all levels of the national TB program, especially when electronic data systems are essential components of the national digital health foundation [12]. Studies have shown that system design barriers adversely impact data quality and data use for decision making [7]. Therefore, it is important to identify the key D2AC measurement dimensions required to cater to the decision-making needs of national TB program stakeholders, ranging from clinical care, laboratory testing, supply chain, and program management to policy development. Understanding data needs, incentives and motivations, skills and knowledge requirements, and technology infrastructure at each health system level and decision stage are foundational for advancing organizational capabilities to transform data to action. Tools are needed that describe interlinkages among systems, people, processes, and institutional capabilities, and that clearly articulate monitoring and improvement indicators for transforming data to action. A D2AC needs to describe a minimum data set; a process for bringing data together, including standards, architectures (data, system, exchange), policies and guidelines, systems, jurisdictions, data sharing, and the benefits resulting from data integration; and risk mitigation and stakeholder roles and responsibilities [13,14]. Even though the proposed D2AC tool is for TB it could be extended to other health programs such as Malaria, HIV, etc., if found to be effective for TB, given that it is being developed using findings about broader HIS.

### Next steps

The literature review is the first step in the development of the D2AC tool. An immediate next step is to develop the tool using the design science approach to maturity models [15]. Maturity models provide a structured way to approach problems by establishing a baseline against which to assess “as is” capabilities and creating a roadmap for improving capabilities to a “desired” level [16]. The development process envisions facilitating an expert review to validate the D2AC tool. A series of virtual workshops, adapting the Delphi method [17], are planned. They will involve a small external group of experts with knowledge and experience in global and country-level TB programs, data use, and HIS projects. Feedback from the virtual workshops will be used to update and implement the D2AC tool with country-level stakeholders, where it will be validated with national TB program stakeholders.

## CONCLUSIONS

Because a country's national TB program functions in the larger and complex health care system, decision-making requirements and factors vary widely. There is a need to develop a validated D2AC tool that clearly articulates the D2AC levels, user roles and associated decision-making requirements, and indicators for each level of the continuum, and which can identify appropriate interventions at different health system levels. Development of D2AC tool should integrate system thinking from the outset to ensure that system design is closer to user reality.

## Additional material


Online Supplementary Document
