# Supplementary material for: Research gaps in transforming tuberculosis data to action for better health outcomes: A systematic literature review
Source: J Glob Health. 2021 Sep 18;11:04058. doi: 10.7189/jogh.11.04058 (PMC8501450; doi:10.7189/jogh.11.04058)
Supplement: Online Supplementary Document [file jogh-11-04058-s001.pdf]

Table S1. Scientific Database Search Terms

Order	Database Name	Search Terms	Number of Results
1	SCOPUS (TITLE-ABS-KEY= Title-Abstract-Keywords)	TITLE-ABS-KEY ("delivery of health care" OR "delivery of healthcare" OR "health care delivery" OR "healthcare delivery" OR "healthcare system" OR "health care system" OR "health system*" OR "healthcare management" OR "health care management") AND TITLE-ABS-KEY ("Health Information System" OR "Health Management Information System" OR "Routine Health Information System" OR "Hospital Information System") AND TITLE-ABS-KEY ("data use" OR "data analysis" OR "data interpretation" OR "data visualization" OR "data accuracy" OR "data quality" OR "decision-making" OR "decision making" OR "evidence-based practice" OR "evidence based practice" OR "communication") AND TITLE-ABS-KEY ("developing country" OR "low resource" OR "limited resource")	122
2	Web of Science Core Collection (TS= Topic)	TS=("delivery of health care" OR "delivery of healthcare" OR "health care deliver*" OR "healthcare deliver*" OR "healthcare system*" OR "health care system*" OR "health system*" OR "healthcare management" OR "health care management") AND TS=("Health Information System*" OR "Health Management Information System*" OR "Routine Health Information System*" OR "Hospital Information System*") AND TS=("data use" OR "data analysis " OR "data interpretation" OR "data visualization" OR "data accuracy" OR "data quality" OR "decision-making" OR "decision making" OR "evidence-based practice*" OR "evidence based practice*" OR "communication*") AND TS=("developing countr*" OR "low resource*" OR "limited resource*")	42
3	PubMed (MeSH-Medical Subject Headings)	("delivery of health care"[MeSH] OR "delivery of healthcare" OR "healthcare deliver*" OR "health care deliver*" OR "healthcare system*" OR "health care system*" OR "health system*" OR "healthcare management" OR "health care management") AND ("Health Information Systems"[MeSH] OR "Hospital Information Systems"[Mesh] OR "Routine Health Information System*" OR "Health Management Information System*") AND ("data use" OR "data analysis"[MeSH] OR "data interpretation" OR "data visualization"[MeSH] OR "data accuracy" [MeSH] OR "data quality" OR "decision-making" OR "decision making" OR "evidence-based practice"[MeSH] OR "evidence based practice*" OR "communication") AND =("developing countr*" OR "low resource*" OR "limited resource*")	56
4	Global Health (TX= All text)	TX ("delivery of health care" OR "healthcare delivery" OR "health care delivery" OR "healthcare system" OR "health care system" OR "health system*" OR "healthcare management" OR "health care management") AND TX ("Health Information Systems" OR "Hospital Information Systems" OR "Routine Health Information System" OR Health Management Information System") AND TX ("data use" OR "data analysis" OR "data interpretation" OR "data visualization" OR "data accuracy" OR "data quality" OR "decision-making" OR "evidence-based practice" OR "communication") AND TX ("developing country" OR "developing countries" OR "low resource" OR "limited resource" OR "low resources")	160
Total			380
References added from two systematic review papers			132
Deduplicated total			411
Papers recommended by experts/networks			5
Total			416

Table S2. Digital Health Repository List

#	Organization Name	Digital Repository Name	Weblink
1	WHO, Geneva, Switzerland	Global Observatory of eHealth	https://www.who.int/goe/en/
2	WHO Regional Office for Africa, Republic of the Congo	Health Situation Analysis	http://aho.afro.who.int/profiles_information/?lang=en
3	Pan American Health Organization, Washington, DC, USA	PAHO Information System for Health	https://www.paho.org/en/information-systems-health-is4h

4	USAID-MEASURE Evaluation, Carolina Population Center, UNC at Chapel Hill, NC, USA	Health Information Systems Strengthening Resource Center	https://www.measureevaluation.org/his-strengthening-resource-center
5	Palladium Group	Health Policy Plus project	http://www.healthpolicyplus.com/

Table S3. List of Peer-Reviewed Publications Selected for Data Extraction

#	Title	Authors	Year Published	Journal	Volume	Issue	Pages
1	Local staff making sense of their tuberculosis data: key to quality care and ending tuberculosis	Heldal E., Dlodlo RA, Mlilo N, Nyathi BB, et al.	2019	The International Journal of Tuberculosis and Lung Disease	23	5	612-618
2	Harnessing the power of data to guide local action and end tuberculosis	Sismanidis C, Shete PB, Lienhardt C, Floyd K, Raviglione M	2017	The Journal of Infectious Diseases	216	suppl_7	S669-S672
3	Understanding the challenges associated with the use of data from routine health information systems in low- and middle-income countries: a systematic review	Hoxha K, Hung YW, Irwin BR, Grépin KA	2020	Health Information Management: Journal of the Health Information Management Association of Australia			
4	Using innovation-decision model to describe the adoption to utilization of HIV-data for decision-making in LMICs	Gesicho MB, Babic A	2018	Studies in Health Technology and Informatics	251		117-120

#	Title	Authors	Year Published	Journal	Volume	Issue	Pages
5	Exploring the information and ICT skills of health professionals in low- and middle-income countries	Koivu A, Mavengere N, Ruohonen MJ, Hederman L, Grimson J	2016	IFIP Advances in Information and Communication Technology	493		152-162
6	Data do count! Collection and use of maternal mortality data in Peru, 1990-2005, and improvements since 2005	Iguiñiz-Romero R, Palomino N.	2012	Reproductive Health Matters	20	39	174-184
7	Improving health information systems for decision making across five sub-Saharan African countries: implementation strategies from the African Health Initiative	Mutale W, Chintu N, Amoroso C, Awoonor-Williams K, Phillips J, Baynes C, Michel C, Taylor A, Sherr K	2013	BMC Health Services Research	13	Suppl 2	S9

#	Title	Authors	Year Published	Journal	Volume	Issue	Pages
8	The interface between the national tuberculosis control programme and district hospitals in Cameroon: missed opportunities for strengthening the local health system - a multiple case study	Keugoung B, Macq J, Buve A, Meli J, Criel B	2013	BMC Public Health	13		265
9	Challenges in moving to "health information for action": an infrastructural perspective from a case study in Tajikistan	Latifov MA, Sahay S	2013	Information Technology for Development	19	3	215-229
10	Embedding health policy and systems research into decision-making processes in low- and middle-income countries	Koon AD, Rao KD, Tran NT, Ghaffar A	2013	Health Research Policy and Systems	11	1	
11	Data-informed decision-making for life-saving commodities investments in Malawi: a qualitative case study	Nemser B, Aung K, Mushamba M, Chirwa S, Sera D, Chikhwaza O, Kachale F	2018	Malawi Medical Journal	30	2	111-119

#	Title	Authors	Year Published	Journal	Volume	Issue	Pages
12	HMIS and decision-making in Zambia: re-thinking information solutions for district health management in decentralized health systems	Mutemwa RI	2006	Health Policy and Planning	21	1	40-52
13	Malaria surveillance and use of evidence in planning and decision making in Kilosa District, Tanzania	Shayo EH	2017	Tanzania Journal of Health Research	19	3	
14	District decision-making for health in low-income settings: a qualitative study in Uttar Pradesh, India, on engaging the private health sector in sharing health-related data	Gautham M, Spicer N, Subharwal M, Gupta S, Srivastava A, Bhattacharyya S, Avan BI, Schellenberg J	2016	Health Policy and Planning	31	Suppl 2	ii35-ii46
15	How to improve local-level data use culture at each level of the health system? An implementation science study	Gashu K, Teklu A, Mancuso A, Tazebew A, Endehabtu B, Mekonnen Z, Tilahun, B	2019	Studies in Health Technology and Informatics	264		1656-1657

#	Title	Authors	Year Published	Journal	Volume	Issue	Pages
16	Perspectives on utilization of community based health information systems in Western Kenya	Flora OC, Margaret K, Dan K	2017	Pan African Medical Journal	27		180
17	Linking macro-level goals to micro-level routines: EHR-enabled transformation of primary care services	Findikoglu M, Watson-Manheim MB	2016	Journal of Information Technology	31	4	382-400
18	Data for decision making: using a dashboard to strengthen routine immunisation in Nigeria	Etamesor S, Ottih C, Salihu IN, Okpani AI	2018	BMJ Global Health	3	5	
19	Assessment of health management information system for monitoring of maternal health in Jaleswar Block of Balasore District, Odisha, India	Dehury RK, Chatterjee SC	2018	Indian Journal of Public Health	62	4	259-264

#	Title	Authors	Year Published	Journal	Volume	Issue	Pages
20	Making health policy management intersectoral: issues of information analysis and use in less developed countries	de Kadt E	1989	Social Science and Medicine	29	4	503-514
21	Routine health information utilization and associated factors among health care professionals working at public health institution in North Gondar, Northwest Ethiopia	Dagnew E, Woreta SA, Shiferaw AM	2018	BMC Health Services Research	18	1	685
22	Information systems for health sector monitoring in Papua New Guinea	Cibulskis RE, Hiawalyer G	2002	Bulletin of the World Health Organization	80	9	752-758
23	Management and use of health information in Malawi and Burkina Faso: the role of technology	Chikumba PA, Ramussen SL	2016	2016 IST-Africa Conference, IST-Africa 2016			

#	Title	Authors	Year Published	Journal	Volume	Issue	Pages
24	Design and implementation of a health management information system in Malawi: issues, innovations and results	Chaulagai CN, Moyo CM, Koot J, Moyo HBM, Sambakunsi TC, Khunga FM, Naphini PD	2005	Health Policy and Planning	20	6	375-384
25	Improving health services to displaced persons in Aceh, Indonesia: a balanced scorecard	Chan GJ, Parco KB, Sihombing ME, Tredwell SP, O'Rourke EJ	2010	Bulletin of the World Health Organization	88	9	709-712
26	Improving quality and use of data through data-use workshops: Zanzibar, United Republic of Tanzania	Braa J, Heywood A, Sahay S	2012	Bulletin of the World Health Organization	90	5	379-384
27	District decision-making for health in low-income settings: a feasibility study of a data-informed platform for health in India, Nigeria and Ethiopia	Avan BI, Berhanu D, Umar N, Wickremasinghe D, Schellenberg J	2016	Health Policy and Planning	31	Suppl 2	ii3-ii11

#	Title	Authors	Year Published	Journal	Volume	Issue	Pages
28	Challenges for health indicators in developing countries: misconceptions and lack of population data	Asah FN, Nielsen P, Saebø JI	2017	IFIP Advances in Information and Communication Technology	504		593-604
29	Production and use of estimates for monitoring progress in the health sector: the case of Bangladesh	Ahsan KZ, Tahsina T, Iqbal A, Ali NB, Chowdhury SK, Huda TM, El Arifeen S	2017	Global Health Action	10	Suppl 1	1298890
30	From data to policy: good practices and cautionary tales	AbouZahr C, Adjei S, Kanchanachitra C	2007	Lancet (London, England)	369	9566	1039-1046
31	Utilization of health information system at district level in Jimma zone Oromia regional state, South West Ethiopia	Abajebel S, Jira C, Beyene W	2011	Ethiopian Journal of Health Sciences	21	Suppl 1	65-76
32	Moving data off the shelf and into action: an intervention to improve data-informed decision making in Côte d'Ivoire	Nutley T, Gnassou L, Traore M, Bosso AE, Mullen S	2014	Global Health Action	7	1	25035

#	Title	Authors	Year Published	Journal	Volume	Issue	Pages
33	Plans and “off-plan activities”: exploring the roles of data and situated action in health planning in Burkina Faso	Rasmussen SL	2018	The Electronic Journal of Information Systems in Developing Countries	84	5	e12049
34	Routine health information system utilization and factors associated thereof among health workers at government health institutions in East Gojjam Zone, Northwest Ethiopia	Shiferaw AM, Zegeye DT, Assefa S, Yenit MK	2017	BMC Medical Informatics and Decision Making	17	116	
35	Health management information system utilization in Pakistan: challenges, pitfalls and the way forward	Qazi MS, Ali M	2011	BioScience Trends	5	6	245-254

#	Title	Authors	Year Published	Journal	Volume	Issue	Pages
36	Confronting challenges in monitoring and evaluation: innovation in the context of the global plan towards the elimination of new HIV infections among children by 2015 and keeping their mothers alive	Radin AK, Abutu AA, Okwero MA, Adler MR, Anyaike C, Asiimwe HT, Behumbiize P, Efuntoye TA, King RL, Kisaakye LN, Ogundehin DT, Phelps BR, Watts H, Weissglas F	2017	Journal of Acquired Immune Deficiency Syndromes	75	Suppl 1	S66-S75
37	Impact of a decision-support tool on decision making at the district level in Kenya	Nutley T, McNabb S, Salentine S	2013	Health Research Policy and Systems	11	1	34
38	Information support for health information management in regional Sri Lanka: health managers' perspectives	Ranasinghe KI, Chan T, Yaralagadda P	2012	The Health Information Management Journal	41	3	20-26
39	The data for decision making project: assessment of surveillance systems in developing countries to improve access to public health information	Wilkins K, Nsubuga P, Mendlein J, Mercer D, Pappaioanou M	2008	Public Health	122	9	914-922

#	Title	Authors	Year Published	Journal	Volume	Issue	Pages
40	Strengthening capacity in developing countries for evidence-based public health: the data for decision-making project	Pappaioanou M, Malison M, Wilkins K, Otto B, Goodman RA, Churchill RE, White M, Thacker SB	2003	Social Science & Medicine (1982)	57	10	1925-1937
41	Exploring how different modes of governance act across health system levels to influence primary healthcare facility managers' use of information in decision-making: experience from Cape Town, South Africa	Scott V, Gilson L	2017	International Journal for Equity in Health	16	1	159
42	Perceptions about data-informed decisions: an assessment of information-use in high HIV-prevalence settings in South Africa	Nicol E, Bradshaw D, Uwimana-Nicol J, Dudley L	2017	BMC Health Services Research	17	Suppl 2	765
43	Improving the use of health data for health system strengthening	Nutley T, Reynolds HW	2013	Global Health Action	6		20001

#	Title	Authors	Year Published	Journal	Volume	Issue	Pages
44	Operational health service management: understanding the role of information in decision-making	Scott V, Dinginto N, Xapile Z	2015	South African Health Review	2014/15		141-150
45	Level of data quality from Health Management Information Systems in a resources limited setting and its associated factors, eastern Ethiopia	Teklegiorgis K, Gebremariam KT, Mirutse G, Lerebo W	2016	South African Journal of Information Management	17	1	
46	Health information as a catalyst for community health system engagement	Colvin CJ, van Pinxteren M, Schmidt BM, Cornell M, Lurie M, Whyle E, Leon N	2018	South African Health Review	2018		135-138
47	District decision-making for health in low-income settings: a systematic literature review	Wickremasinghe D, Hashmi IE, Schellenberg J, Avan BI	2016	Health Policy and Planning	31	Suppl 2	ii12-ii24

#	Title	Authors	Year Published	Journal	Volume	Issue	Pages
48	District decision-making for health in low-income settings: a case study of the potential of public and private sector data in India and Ethiopia	Bhattacharyya S, Berhanu D, Taddesse N, Srivastava A, Wickremasinghe D, Schellenberg J, Avan BI	2016	Health Policy and Planning	31	Suppl 2	ii25-ii34

Table S4. List of Grey Literature

#	Author	Year	Title
1	Palladium, Health Policy Plus	2019	Total market approach projection tool: user's guide and lessons learned from an application in two countries
2	MEASURE Evaluation	2018	Performance of routine information system management (PRISM) user's kit - moving from assessment to action
3	MEASURE Evaluation	2018	Routine health information system rapid assessment tool
4	WHO	2018	Analysis and use of health facility data: guidance for tuberculosis programme managers
5	WHO	2019	Analysis and use of health facility data: guidance for national and district planners and managers
6	WHO	2018	Analysis and use of health facility data: general principles
7	WHO	2014	Understanding and using tuberculosis data
8	Stop TB Partnership	2017	Data for action for tuberculosis key, vulnerable and underserved populations
9	WHO	2018	Cascade data use manual: to identify gaps in HIV and health services for programme improvement
10	WHO	2019	People-centered framework for TB programme planning and prioritization: user guide
11	Ministry of Health - El Salvador, Health Policy Plus	2019	Sustainability roadmap for multisectoral action to address tuberculosis in El Salvador
12	Palladium, Health Policy Plus	2019	El Salvador's response for tuberculosis control: a sustainability analysis
13	Futures Group, Health Policy Project	2012	Linking health policy with health systems and health outcomes: a conceptual framework
14	Futures Group, Health Policy Initiative, Task Order 1	2010	The art of moving from policy to action: lessons learned from the USAID Health Policy Initiative (2005-2010)
15	USAID – Broadreach| Regional Action through Data	n.d.	Data-driven strategy to improve health outcomes in sub-Saharan Africa
16	Foundation for Innovative New Designs - WHO Collaborating Center	n.d.	Case study: FIND: Be data driven FIND's Actionable Diagnostic Data for improved TB CARE (ADD for TB) initiative
17	KNCV Tuberculosis Foundation	n.d.	Making sense of TB data: guide for collection, analysis and use of TB data for health workers in Zimbabwe
18	Knowledge, Evidence and Learning Programme - UK AID - DFID	2018	Designing and implementing health management information systems
19	Jaako Yrjö Koskinen Thesis	2012	The influence of organizational culture on the use of routine health information in decision making within government health services in Rural Burkina Faso
20	USAID - Challenge TB	n.d.	Scorecard Ethiopia
21	USAID - Challenge TB	2015 baseline	Scorecard Indonesia
22	USAID - Challenge TB	2017-2018	Scorecard Zambia

Table S 5. List of HIS Assessment Tools

1. HIS Stages of Continuous Improvement (SOCI) Toolkit

2. Health Information Systems Interoperability Maturity Toolkit (IMM)
3. WHO SCORE (Survey, Count, Optimize, Review, Enable)
4. PRISM Tools for Community Health Information Systems

Table S6. Illustrative List of Data Use Barriers (from peer reviewed literature [*n* = 48])

**Author**	**Date**	**Type of Study**	**Country**	**Level of Healthcare System**	**Reported Data Use Barriers**
Richard I. Mutemwa	2006	Case Study	Two districts in Zambia	District	Lack of trust in quality data; lack of understanding and skills to interpret and use data; poor information product; lack of accountability for accurate data; slow timeliness and feedback on information; poor information use culture
Tara Nutley, Léontine Gnassou, Moussa Traoré, Edwige Abitche-Bosso, and Stephanie Mullen	2014	Other	Côte d'Ivoire	General	Low data quality, which decreases demand for data; weakness in availability of health data at all levels; insufficient number of skilled M&E professionals (especially at the regional level)
Patrick A. Chikumba and Stine L. Rasmussen	2016	Other	Malawi and Burkina Faso	National	Data collection and dissemination were not strongly supported
Richard E. Cibulskis and Gilbert Hiawalyer	2002	Other	Papua New Guinea	General	Haphazard information systems made it difficult to aggregate data on a national level, compare results between provinces, or maintain systems
Ranjit Kumar Dehury and Suhita Chopra Chatterjee	2018	Other	Balasore District, Odisha, India	General	Lack of a trained workforce; substandard M&E; poor data quality; inadequate data collection tools; poor Internet connectivity
Annariina Koivu, Nicholas Mavengere, Mikko. J. Ruohonen, Lucy Hederman, and Jane Grimson	2016	Review	LMICs	General	Computer illiteracy; lack of trained healthcare workers in routine HIS; lack of skills in data security, data management, and data analysis
Murodillo Abdusamadovich Latifov and Sundeep Sahay	2013	Case Study	Tajikistan	National	Rampant redundancies in HIS; health management information system (HMIS) data-driven not action-led; extreme reluctance to change
Leonard E.G. Mboera, Susan F. Rumisha, Tabitha Mlacha, Benjamin K. Mayala, Veneranda M.Bwana, and Elizabeth H. Shayo	2017	Other	Kilosa District, Tanzania	Facility	Poor data management; delayed and inefficient reporting; shortage of data collection and reporting tools; lack of data analysis capacity and overburdened health staff; weak communication systems; weak capacity for facility-level decision making; multiple surveillance systems

Table S7. Illustrative List of Data Use Enablers (from peer reviewed literature [*n* = 48])

**Author**	**Date**	**Type of Study**	**Country**	**Level of Healthcare System**	**Data Use Enablers**
Sultan Abajebel, Challi Jira, and Waju Beyene	2011	Other	Ethiopia	General, community, district	Data collection is in alignment with the workflow

Author	Date	Type of Study	Country	Level of Healthcare System	Data Use Enablers
Flora N. Asah, Petter Nielsen, and Johan I. Sæbø	2017	Review	Cameroon	General, facility, community, district, regional/provincial, national	Established analytics and visualizations; increased data quality; increased decision-support mechanisms
Sanghita Bhattacharyya, Della Berhanu, Nolawi Taddesse, Aradhana Srivastava, Deepthi Wickremasinghe, Joanna Schellenberg, and Bilal Iqbal Avan	2016	Case Study	India and Ethiopia	General, community, district	Quality data that encompass all attributes of the health system
Chet N. Chaulagai, Christon M. Moyo, Jaap Koot, Humphrey B. M. Moyo, Thokozani C. Sambakunsi, Ferdinand M. Khunga, and Patrick D. Naphini	2005	Review	Malawi	Facility	Presence of data systems training in academic settings and built into job descriptions, thus increasing health data literacy; strong data governance presence
Muhammad Suleman Qazi and Moazzam Ali	2011	Other	Pakistan	General, facility, district, regional/provincial, national	Increase in data quality; quick data transmission; regular and timely feedback to facilities; use of HMIS at community level
Kaduruwane I. Ranasinghe, Taizan Chan, and Prasad Yaralagadda	2012	Other	Sri Lanka	Regional/provincial	Increased decision support operations
Stine L. Rasmussen	2018	Case Study	Burkina Faso	District	Situated action used to create daily order in real time (e.g., practical norms), allowing most activities to be done despite delays
Vera Scott, Ntombomzi Dinginto, and Zethu Xapile	2015	Case Study	South Africa	Facility	Robust standard operating procedures; routine review of facility reports

Table S8. Data Use Barriers and Enablers (from grey literature [*n* = 22])

Author	Date	Paper Type	Country	Level of Healthcare System	Data Use Barriers
Knowledge for Development (K4D)/United Kingdom	2018	Review	Global	Not explicitly mentioned	Barriers to sharing routinely collected public data: information and communications technology (ICT) and parallel reporting; motivational

Department for International Development (DFID) funded					barriers; economic barriers; political, legal, and ethical barriers
					**Enablers**
Measure Evaluation	2018	Guidelines	Global	National	Stakeholder engagement; a matrix for facilitating prioritization of interventions along a continuum of easy-to-hard to implement and impact information system performance (low to high); a planning matrix for organizing the implementation of interventions
Measure Evaluation	2018	Guidelines	Global	All levels	Identification of data use barriers and suggested interventions to overcome them
K4D/DFID funded	2018	Review	Global	Not explicitly mentioned	Enabler for implementation: leadership motivation and regular feedback; donor support and collaboration

Table S9. Relationship Among Decision Type, Stakeholders Involved, and Data Source

**Decision Made**	**Stakeholders Involved in Decision**	**Data Source**	**Author**
Strategic/Policy	Community health center workers at subdistrict level; subcenter facility and community-level workers at the community level	Health infrastructure information system	Sanghita Bhattacharyya, Della Berhanu, Nolawi Taddesse, Aradhana Srivastava, Deepthi Wickremasinghe, Joanna Schellenberg, and Bilal Iqbal Avan
Operational	District offices	Health facility assessments	Sultan Abajebel, Challi Jira, and Waju Beyene
Clinical	Family medicine personnel (e.g., physicians, nurses, and midwives)	Community/service records	Melike Findikoglu and Mary Beth Watson-Manheim
Program	Health staff	Health infrastructure information system	Christopher J. Colvin, Bey-Marrie Schmidt, Myrna van Pinxteren, Morna Cornell, Eleanor Whyle, Mark Lurie, and Natalie Leon
Clinical	Primary care level clinical staff	District and health facility assessments	Bilal Avan, Della Berhanu, Nasir Umar, Deepthi Wickremasinghe, and Joanna Schellenberg
Not explicitly mentioned	Central Monitoring and Evaluation Division	Health facility assessments	Patrick A. Chikumba and Stine L. Rasmussen
